# Anterolateral Thigh Flap and Bone Plate for Mandibular Reconstruction in Patients over 55 Undergoing Ablative Oral Surgery: A Systematic Review and Meta-Analysis

**DOI:** 10.3390/jcm15093457

**Published:** 2026-05-01

**Authors:** Riccardo Nocini, Giacomo Papi, Giulia Gobbo, Athena Eliana Arsie, Gianluca Colapinto, Funda Goker, Matteo Seno, Valerio Arietti, Massimo Del Fabbro

**Affiliations:** 1 Unit of Otolaryngology, Head and Neck Department, University of Verona, 37134 Verona, Italy; riccardo.nocini@aovr.veneto.it (R.N.); papi.giacomo@outlook.it (G.P.); giulia.gobbo@univr.it (G.G.); athena.arsie@gmail.com (A.E.A.); matteoseno98@gmail.com (M.S.); valerio.arietti@aovr.veneto.it (V.A.); 2 Unit of Maxillo Facial Surgery, Head and Neck Department, University of Verona, 37134 Verona, Italy; gianluca.colapinto@unimi.it; 3 Department of Biomedical, Surgical and Dental Sciences, University of Milan, 20122 Milan, Italy; funda.goker@unimi.it; 4 UOC Maxillofacial Surgery and Dentistry, Fondazione IRCCS Ca’ Granda Ospedale Maggiore Policlinico, 20122 Milan, Italy; 5 Department of Oral and Maxillofacial Surgery, Faculty of Dentistry, Istanbul Aydın University, 34295 Istanbul, Türkiye

**Keywords:** anterolateral thigh flap, bone plate, bone reconstruction, elderly patients, mandibular reconstruction, head and neck cancer, oral cancer, meta-analysis, systematic review

## Abstract

**Background/Objectives**: Mandibular reconstruction following ablative oral surgery is particularly challenging in elderly patients due to comorbidities and reduced physiological healing potential. While vascularized bone flaps represent the gold standard, the combination of an anterolateral thigh (ALT) flap and a bone reconstruction metallic plate may be considered as a less invasive alternative. This systematic review aimed to evaluate the effectiveness of such reconstructive techniques, in terms of success rate and complication rate, in patients aged over 55. **Methods**: Studies were included if the sample size consisted of at least 20 patients undergoing mandibular reconstruction with an ALT flap and plate following oral cavity resection for benign or malignant conditions. Studies were excluded if relevant outcomes were not reported and the mean age was <55 years. An electronic search was conducted in PubMed, Scopus, Web of Science and Embase. The last search was made on 26 February 2026. Data extracted included patient demographics, clinical outcomes and postoperative plate-related and overall complications. Risk of bias assessment was undertaken using the Joanna Briggs Institute tool for cohort studies and case series. Proportional meta-analysis was conducted to estimate the overall clinical success and the complication rate. **Results**: Of the 525 studies initially screened, four studies including a total of 329 patients (292 males, 37 females) with an overall mean age > 55 years were included. Mean hospital stay ranged from 10 to 24 days. The overall clinical success rate of the ALT flap procedure was 97% (95% confidence intervals (CIs): 92%, 99%). Flap-related complications were rare, with flap loss reported in only one patient and partial necrosis or failure affecting up to 6.6% of cases. Conversely, the overall plate-related complications rate was 28% (95% CI: 15%, 41%), with plate exposure rates ranging from 10% to 38.7% in the included studies. Wound complications, including infection and fistula formation, ranged from 20% to 38.7% of patients. **Conclusions**: In patients over 55, despite the not negligible rate of complications, the use of ALT flaps and reconstruction plate represents a viable alternative to vascularized bone flaps for mandibular reconstruction, particularly when comorbidities or frailty preclude more complex procedures. Further studies with a large sample size are needed to validate these findings and guide clinical decision-making.

## 1. Introduction

Mandibular reconstruction for segmental defects remains a fundamental challenge in head and neck surgery, particularly among elderly patients. The mandible is essential for mastication, speech, swallowing, and maintaining the lower facial contour and aesthetics. Consequently, mandibular defects resulting from ablative procedures can significantly impair functional abilities and psychological well-being, ultimately compromising nutritional status, communication, social integration, and overall quality of life [1,2].

Microsurgical free tissue transfer has become the standard of care for reconstructing extensive mandibular defects, offering superior outcomes compared to non-vascularized grafts and regional flaps. The vascularized fibula free flap (FFF), first introduced by Hidalgo in 1989, provides robust bone stock and reliable vascularity, enabling both structural and functional restoration as well as potential for dental rehabilitation [3,4].

In recent years, numerous bone flaps for mandibular reconstructions have been described in the literature, underlining the possible complications also at the donor-site level [5,6]. A meta-analysis of 24 studies published in 2016 reported that donor-site complications are more frequent using fibula free flaps compared to iliac crest, while iliac crest flaps are associated with more delayed healing and suture failure at the recipient site [7]. A more recent meta-analysis based only on reports from double-arm studies concluded that there are no significant differences between these two flap approaches regarding the odds ratio of failures (*p* = 0.563), donor-site complications (*p* = 0.756), and recipient-site complications (*p* = 0.452) [8].

Despite the advantages, FFF reconstruction is a technically demanding procedure, often involving prolonged operative times and considerable donor-site morbidity. These factors are particularly relevant in elderly patients, who frequently present with age-associated comorbidities, reduced physiological reserve, and impaired wound healing, all of which can increase perioperative risk and complicate postoperative recovery [9,10,11].

In contrast, the anterolateral thigh (ALT) flap combined with a titanium reconstruction plate has emerged in recent decades as a less invasive alternative, particularly suited for patients with elevated surgical risk. First introduced by Koshima et al. in 1993, the ALT flap provides wide soft tissue coverage, has a reliable vascular pedicle, and is associated with low donor-site morbidity [12]. Although this approach avoids the morbidity of bone harvesting and can shorten operative time, its reliance on alloplastic materials introduces potential long-term complications such as plate exposure, fracture, or infection, which may necessitate revision procedures [13,14,15]. A retrospective study by Fanzio et al. on 130 patients undergoing mandibular reconstruction with ALT flap and plate reported a 5-year plate exposure-free rate of 32.8% and 64.3% for patients with and without postoperative radiotherapy, respectively, with a global plate exposure incidence as high as 37.7% [14]. Such complications have been partly related to the shape, extent and location of the mandibular defects [14]. A recent retrospective case series by Ottaviano et al. on mandibular reconstruction with ALT free flap and bridging plate in 34 oncological patients followed up to 7 years reported a lower but not negligible incidence of plate-related complications, with plate exposure in five cases (14.7%) and plate fracture in four cases (11.8%) [15].

Elderly individuals pose unique reconstructive challenges due to multifactorial vulnerabilities including frailty, polypharmacy, cognitive decline, and nutritional deficiencies. These considerations call for a surgical strategy that balances functional outcomes with procedural risk, aiming to preserve quality of life while minimizing invasiveness and morbidity [11].

Given this clinical context, the ALT flap with plate reconstruction may offer an advantageous reconstructive strategy in appropriately selected elderly patients, despite a major risk of complications, especially plate-related ones. To investigate this hypothesis, we performed a systematic review focusing on mandibular reconstruction in patients aged ≥55 years. Our objective was to evaluate clinical outcomes, along with postoperative complication rate, associated with the use of the ALT flap with titanium reconstruction plate in the elderly population, addressing a critical gap in the literature where older patients are often under-represented or not specifically analyzed as a distinct cohort.

## 2. Materials and Methods

A systematic literature search was conducted using the PubMed, Scopus and Web of Science databases to identify relevant studies reporting on the use of the ALT flap combined with a reconstruction plate for mandibular reconstruction in the context of oral cavity surgery. This systematic review was conducted in accordance with the Preferred Reporting Items for Systematic Reviews and Meta-Analyses (PRISMA) guidelines [16]. The review was registered in the PROSPERO database (registration number: CRD420251131163).

### 2.1. PICO Question

In patients aged ≥ 55 years undergoing mandibular reconstruction using the anterolateral thigh flap combined with a titanium reconstruction plate, what are the success rates, the postoperative complication rates and types?

P (population): Patients aged at least 55 years who underwent mandibular bone resection due to oral or head and neck cancer;

I (intervention): Mandibular reconstruction using ALT flap and titanium reconstruction plate;

C (comparison): Since the review aimed at evaluating success and complications associated with a specific reconstructive treatment, comparative treatments were not considered;

O (outcomes): Primary outcomes were clinical success rate and complication rate; secondary outcomes were complication type and the duration of hospital stay.

### 2.2. Search Strategy

An electronic search was conducted through the PubMed, Scopus, Web of Science and Embase databases. The search string used in each database is shown in Table 1.

No temporal restrictions were applied to the literature search, which was last conducted on 26 February 2026. No gray literature search was undertaken. The reference list of the included studies and systematic reviews was also assessed for possible eligible studies.

### 2.3. Eligibility Criteria

Studies were eligible for inclusion if they met all of the following criteria: (I) studies involving at least 20 patients; (II) patients mean age ≥ 55 years; (III) mandibular reconstruction was performed following mandibulectomy due to either benign or malignant pathology; (IV) an anterolateral thigh flap was used in combination with a reconstruction plate; (V) details on success and complications were reported.

The exclusion criteria were as follows: (I) case reports, conference papers, letters, editorials, and book chapters; (II) studies not involving human subjects; (III) studies that did not report the use of a reconstruction plate; (IV) articles not published in the English language; (V) studies with a mean age of patients less than 55 years; (VI) narrative or systematic reviews or meta-analyses.

### 2.4. Screening

All the retrieved studies were initially screened by two independent reviewers (RN and GP) based on titles and abstracts. Then, the full text of all the eligible studies was obtained and carefully examined to confirm if the studies met the selection criteria. Any disagreement was resolved via discussion between the two reviewers or consulting with a third reviewer (MDF). The level of agreement between the reviewers regarding study inclusion was estimated using Cohen’s kappa coefficient. Data regarding patient and treatment features, as well as clinical outcomes, were independently extracted from all the included studies by the same two reviewers using a predetermined data extraction form. In case of missing information, the reviewers contacted the study authors to get the required data. If no or inconclusive feedback was obtained, the data were excluded from the analysis.

### 2.5. Risk of Bias

Two co-authors (GG, GC) independently evaluated the included studies for risk of bias. A third reviewer was consulted (MDF) in case of doubts or discrepancies. For nonrandomized, cohort studies, the risk of bias was assessed using the Joanna Briggs Institute (JBI) Critical Appraisal checklist for cohort studies, while the JBI checklist for case series was used in the case of non-comparative studies (https://jbi.global/critical-appraisal-tools, accessed on 29 August 2025). For randomized controlled trials (RCTs), the Cochrane Risk of Bias assessment tool (RoB 2.0) was used. The JBI checklist for cohort studies covers eleven different domains, and the one for case series covers 10 domains. The potential risk levels were judged as low, moderate, serious, or critical. The studies were classified at critical risk of bias if one or more items were critical, at serious risk if more than 4 items were unclear and none critical, at moderate risk if 2 to 4 items were unclear, and at low risk if there was no or one item judged unclear. The Cochrane RoB 2.0 tool consists of seven distinct bias categories (random sequence generation, allocation concealment, blinding of participants and personnel, blinding of outcome assessment, incomplete outcome data, selective reporting, and other bias). RCTs were judged at high risk of bias if at least one of the domains had a high risk of bias or if more than two domains presented some concerns, and at low risk if all the domains were at low risk of bias; in all the other cases, the study was considered to have some concerns about risk of bias.

### 2.6. Data Synthesis and Analysis

It was planned to perform a meta-analysis of proportions for each outcome considered (ALT flap failures and complications) if at least three studies were available to give an overall view of the clinical effectiveness and complication rate of the reconstructive treatment. In case studies comparing different flap types were included, only the arm using the ALT flap was considered. Given the low number of studies and the substantial heterogeneity across studies, a random effects model was used. Due to the dichotomous nature of data, the DerSimonian and Laird inverse variance method was used to minimize the imprecision (uncertainty) of the pooled effect estimate [17]. For single-arm proportion meta-analysis, the logit transformation was applied when proportions were outside the 0.2–0.8 range (e.g., in the case of flap success) to stabilize variance and normalize data. Raw data were used, without applying any transformation, for proportions between 0.2 and 0.8. Statistical heterogeneity (total variation in effect estimates that was due to heterogeneity across studies rather than sampling error (chance)) was estimated using I^2^ statistics. A *p*-value of 0.10 was used to determine significance in statistical heterogeneity [17]. The thresholds defined in the Cochrane Handbook for Systematic Reviews of Interventions were considered for the interpretation of the I^2^ statistic [17]. In general, if I^2^ is <40% the heterogeneity may not be important, for I^2^ from 30% to 60% it is moderate, if I^2^ is from 50% to 90% there is a substantial heterogeneity, while for I^2^ values 75–100% there is considerable heterogeneity [17]. As acknowledged in the same Cochrane Handbook, these thresholds defined for meta-analysis of randomized trials should be interpreted cautiously, since the relevance of inconsistency across studies may depend on several factors. Prediction intervals were estimated to present the extent of between-study variation. It was decided that, if there was high heterogeneity in study protocols, materials and patients’ characteristics, which made data aggregation from at least three studies unfeasible, no meta-analysis would be performed, and the results would be presented in a narrative way. The software StataNow Version 19 MP (StataCorp LLC, College Station, TX, USA) and the online tool MetaAnalysisOnline (www.metaanalysisonline.com) were used for meta-analysis.

The certainty of evidence for the main outcomes considered in this review (flap success rate, plate-related complication rate and overall complication rate) was estimated using the GRADE approach and presented using a standard summary of findings table (epoc.cochrane.org/resources/epoc-resources-review-authors (accessed 2 April 2026)).

## 3. Results

**Figure 1:**
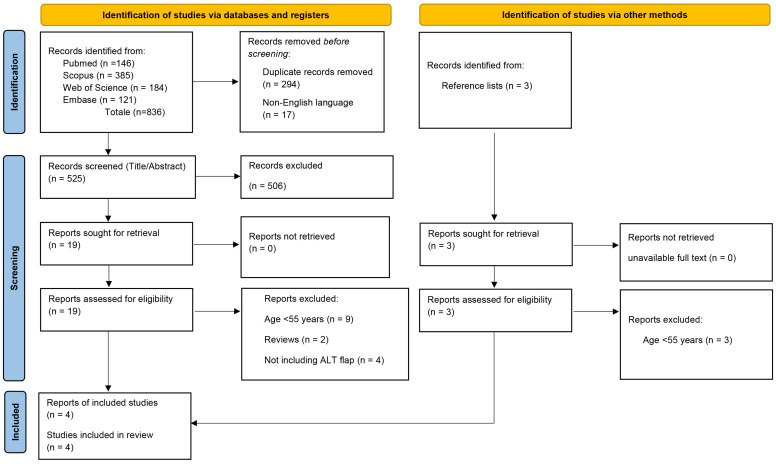
PRISMA 2020 flow diagram for systematic reviews, which included searches of databases and other sources.

A total of 836 records were initially identified through database searching. After removing 294 duplicates, 17 non-English language articles were excluded prior to screening. The remaining 525 records were assessed based on title and abstract by three independent reviewers (G.P., G.G., G.C.). Of these, 19 articles were selected for full-text evaluation. An additional three articles were identified through manual screening of the reference lists of the full-text articles. After full-text assessment, only four studies met the eligibility criteria and were included in the final analysis [18,19,20,21]. The full study selection process is illustrated in the PRISMA 2020 flow diagram (Figure 1).

In Table 2 the demographic and clinical characteristics of the patient population are reported for each included study. In Table 3 the postoperative complications are summarized in detail.

Collectively, the four included studies reported data on 329 patients, with a total of 292 male and 37 female patients [18,19,20,21]. All the studies had a retrospective design and only the study by Bowe et al. [19] reported on a single cohort of patients, while the other studies were comparative.

### 3.1. Risk of Bias

**Figure 2:**
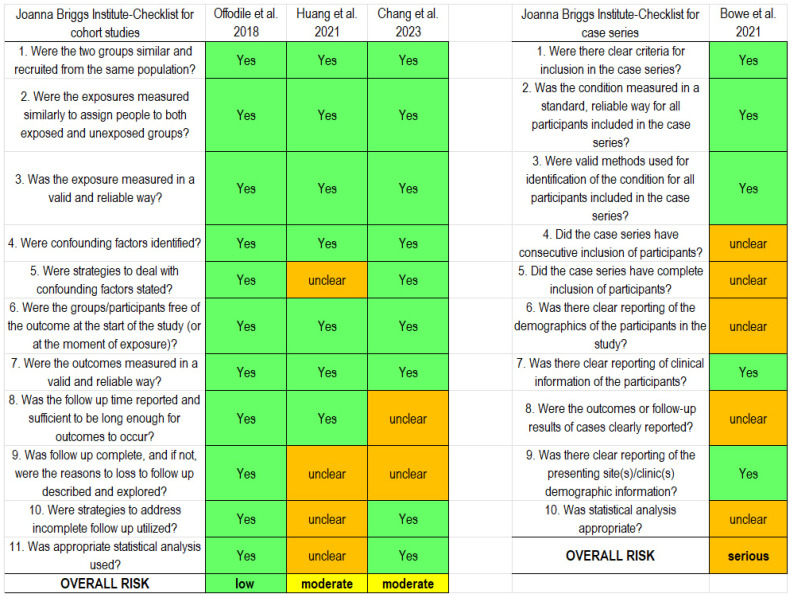
Risk of bias assessment for the three comparative cohort studies (**left panel**) [18,20,21] and for the retrospective case series study (**right panel**) [19].

The results of the risk of bias assessment are summarized in Figure 2. The only study at low risk of bias was the one by Offodile et al. (2018) [18], while according to the JBI checklist for cohort studies, Huang et al. (2021) [20] and Chang et al. (2023) [21] were judged at moderate risk. The study by Bowe et al. (2021) [19] was evaluated with the JBI checklist for case series and was judged at serious risk of bias.

### 3.2. Summary of the Features of Included Studies

Offodile et al. [18] conducted a retrospective, propensity score-matched study comparing the long-term outcomes of oromandibular defect reconstruction using an ALT flap with a bridging plate versus simultaneous reconstruction with soft tissue and a vascularized bone flap. The unmatched ALT group included 127 patients (120 males and seven females) with a median age of 55.9 ± 12.3 years. Following propensity score matching, 31 patients (29 males and two females) were selected for comparative analysis against a group of 31 patients undergoing double flap (soft tissue and vascularized bone flap). Only the former group was considered for the present review. The mean hospital stay in the ALT group was 23.9 ± 11.6 days, and the follow-up was at least six months. No flap loss was reported in the entire series of ALT patients. Nevertheless, four patients underwent re-exploration (12.9%), and 12 patients (38.7%) suffered from wound infection or fistula formation. The plate exposure rate was also 38.7%, with one patient (3.2%) experiencing a plate fracture. This study also assessed postoperative quality of life based on a questionnaire administered at least 12 months after surgery. Of the items evaluated, only the pain score resulted significantly lower in the ALT flap + plate group, compared to the double flap group (*p* < 0.001).

Bowe et al. [19] in 2021 reported the results of a single cohort of 30 patients with lateral posterior segmental mandibular defects treated with bridging reconstruction plate and anterolateral thigh free flap, including 19 males and 11 females, with a mean age of 67 years (range: 31 to 87 years). Most patients were classified as ASA class I or II, and the median length of hospital stay was 10 days. The mean follow-up was 23.3 months (range: 1 to 96 months). Twenty-eight patients underwent oncological ablative surgery, while two required reconstruction for osteoradionecrosis affecting the posterior mandible. The overall reconstruction-specific complication rate was 15.3% (n = 4). Flap loss occurred in only one patient, resulting in a flap success rate of 96.7%. Additionally, three patients experienced plate-related complications, including two cases of infection and one case of plate extrusion.

Huang et al. [20] compared post-reconstructive events in patients undergoing ablative surgery for a malignant tumor and subsequent primary reconstruction with ALT flap or primary fibula flap and reconstruction plate versus those requiring secondary fibula flap transfer due to complications. A total of 114 patients (105 males and nine females) with a median age of 56.3 ± 10 years underwent reconstruction using a plate and ALT flap in a single Hospital and were considered for the present review. The follow-up period was 16.0 ± 11.6 months (range: 3 to 55 months). Twelve patients ultimately required a secondary fibula flap due to plate exposure. Among plate-related complications, the overall plate exposure rate was 27% (31 out of 114 patients). Other post-reconstructive events regarded surgical debridement (46/114 patients) and screw loosening for patients followed longer than 12 months (21/52 patients).

Chang et al. [21] in 2023 published a study focused on plate-related complications and quality of life of patients undergoing mandibulectomy and reconstruction, comparing outcomes between fibula flaps with reconstruction plate (n = 86 patients), fibula flap with miniplate (n = 61), and ALT flap with reconstruction plate (n = 58). Only the latter group was considered for this review. The patients were predominantly male (56 males and two females), with a median age of 56.8 years. Most patients presented with comorbidities such as cardiovascular disease, diabetes, or liver disease, and many had a history of smoking and/or alcohol use. Reconstruction was indicated for malignancy in 57 patients (98.3%) and in one case for osteoradionecrosis. No patients underwent surgery for benign conditions. The median hospital stay was 19.5 days. Postoperative complications included partial flap necrosis in four patients and flap failure in three patients, with four patients requiring revision surgery. Plate-related complications were more frequent in the ALT group than in the fibula group, occurring in 22 patients (37.9%). The most common issues were plate exposure (24.1%), plate fracture or dislodgement (24.1%), plate infection (20.7%), and oral fistula formation (31%). Postoperative radiotherapy and osteonecrosis negatively affected the occurrence of complications. Finally, it was found that plate-related complications were associated with worse pain scores, negatively affecting the patients’ quality of life.

### 3.3. Meta-Analysis Results

No pairwise meta-analysis was performed because the comparison treatments differed between the studies, and the review was primarily focused on reconstruction using ALT flaps with titanium plates.

**Figure 3:**
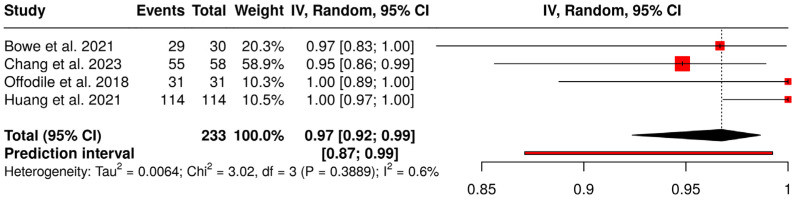
Forest plot showing proportional meta-analysis of flap success. Logit transformation was applied to data [18,19,20,21].

A proportional meta-analysis of success rates is shown in Figure 3. Four studies were analyzed with a total of 233 subjects. Based on the analysis performed using a random effects model with the inverse variance method and logit transformation, the summarized proportion is 0.97 (95% CI: 0.92; 0.99). No significant heterogeneity across studies was detected (*p* = 0.39, I^2^ = 0.6%, 95% CI for I^2^: 0; 0.848), suggesting that the effect sizes across studies were consistent in both magnitude and direction. The prediction interval (0.87 to 0.99) suggests a good prognosis for the investigated surgical technique.

**Figure 4:**
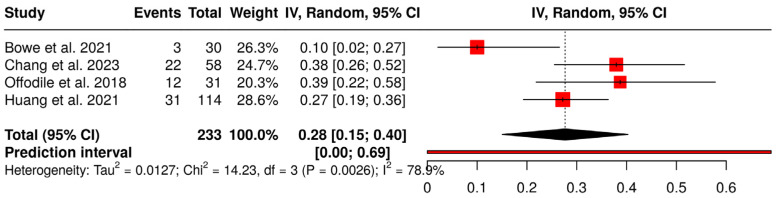
Forest plot showing proportional meta-analysis of plate-related complication rate [18,19,20,21].

Regarding plate-related complications, the proportional meta-analysis shown in Figure 4 found an overall complication rate of 28% (95% CI: 15%; 40%). A significant heterogeneity was detected (*p* < 0.01), suggesting inconsistent effects in magnitude and/or direction. The I^2^ value indicates that 79% of the variability among studies arises from heterogeneity rather than random chance (95% CI for I^2^: 0.436; 0.921). The prediction interval confirms a very high variability for this outcome. Looking at the plot, it appears that the heterogeneity is mainly due to the study by Bowe et al. [19] which had a serious risk of bias. A sensitivity analysis done by excluding the Bowe et al. study [19] yielded a plate-related complication rate of 33% (95% CI: 25%; 40%), with no significant heterogeneity among studies (*p* = 0.25, I^2^ = 28%, 95% CI for I^2^: 0; 0.925).

**Figure 5:**
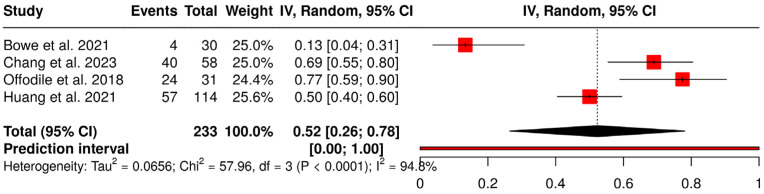
Forest plot showing proportional meta-analysis of overall complication rate [18,19,20,21].

Considering the total number of complications reported in each study, the proportional meta-analysis shown in Figure 5 found an overall complication rate of 52% (95% CI: 26%, 78%). A significant heterogeneity was detected (*p* < 0.01), suggesting inconsistent effects in magnitude and/or direction. The I^2^ value indicates that 95% of the variability among studies arises from heterogeneity rather than random chance (95% CI for I^2^: 0.898; 0.974). The very wide prediction interval confirms a large variability for this outcome. Also in this case, such heterogeneity appears mainly due to the study by Bowe et al. [19] which reported a very low proportion of complications, as opposed to the other studies. The sensitivity analysis done by excluding the Bowe et al. study [19] yielded a total complication rate of 65% (95% CI: 48%, 81%), with still a significant heterogeneity among studies (*p* = 0.0025, I^2^ = 83%, 95% CI for I^2^: 0.496; 0.945).

Supplementary Table S1 reports the results of the assessment of the certainty of evidence for the outcomes considered in this review as a summary of findings table. For all the outcomes the certainty was scored as very low, suggesting that, given the multiple weaknesses of the available evidence, this review only provides some indication of the likely effect. However, the likelihood that the true effect will be substantially different is very high.

## 4. Discussion

This systematic review evaluated the use of the anterolateral thigh (ALT) flap combined with a reconstruction plate for mandibular reconstruction in patients aged 50 years and older following ablative surgery of the oral cavity. Currently, the literature is lacking in studies that focus exclusively on the use of the anterolateral thigh (ALT) flap in combination with a reconstruction plate for mandibular reconstruction following ablative surgery, whether for oncological or non-oncological reasons. A high number of studies analyzing various reconstructive techniques are present in the literature [22,23,24,25,26,27,28,29,30,31]; however, these studies often lack specific demographic, clinical, and postoperative data for each individual technique, or include patients with a wide age range and highly heterogeneous baseline clinical conditions. This makes it difficult to extract data specifically concerning patients who underwent mandibular reconstruction with ALT and plate, especially when attempting to identify only elderly patients with comorbidities, which represented the focus of the present review. Despite the limited number of studies and patients, Bowe et al. in 2021 suggested that this reconstructive strategy can be a viable alternative to vascularized bone flaps in this specific population, with only 10% of plate-related complications [19], consistent with those published on the outcomes from osseous flap-based mandibular reconstruction between 10.9% and 13.7% [32,33]. However, it must be considered that the Bowe et al. study [19] was found to be at high risk of bias, and its highly encouraging findings should be interpreted cautiously.

Vascularized bone flaps, particularly the fibula free flap, are widely regarded as the gold standard for mandibular reconstruction due to their ability to restore both form and function, including the potential for dental rehabilitation [3,4,30]. However, these procedures are technically demanding, require longer operative times, and may carry higher perioperative risks, which can be especially challenging for elderly patients with multiple comorbidities [22]. In contrast, the use of soft tissue flaps such as the ALT combined with a reconstruction plate offers a less complex alternative, with shorter operative times and reduced donor-site morbidity [23,31]. Importantly, Chepeha et al. (2008) [24], Arden et al. (1999) [34], Day et al. (2014) [35] and Deleyiannis et al. (2006) [36] outlined clear indications for using a bridging reconstruction plate combined with a soft tissue free flap. These include cases where bony reconstruction is contraindicated due to systemic health limitations, patients unable to sustain prolonged surgery, prior radiation therapy, patient choice (particularly when dental rehabilitation is not desired nor appropriate), and anatomical complexity (such as ablative defects that have a large reconstructive requirement for soft tissue that are less suited to fibular osteo-cutaneous flaps). In the last case, soft tissue flaps restore contour and provide adequate coverage for hardware while minimizing operative risk [24,34,35,36]. However, plate-related complications remain a significant concern, as demonstrated in this review, and are probably the most critical issue of this type of surgery. This was also described by Chang et al. (2023), who underlined the extremely negative impact of some risk factors such as osteoradionecrosis and postoperative radiotherapy [21]. Several studies have demonstrated that the absence of bony support in soft tissue flap and plate reconstruction increases the risk of plate exposure. Blackwell et al. (1996) reported a notably higher rate of plate exposure in patients reconstructed with soft tissue free flaps and plates compared to those receiving vascularized bone flaps [25]. This vulnerability is primarily attributed to increased mechanical stress on the hardware and compromised soft tissue coverage, particularly in irradiated or previously operated fields [25]. This finding is reinforced by the systematic review and meta-analysis conducted by Bauer et al. (2020), which showed that soft tissue flap and plate reconstructions are associated with significantly higher rates of plate extrusion and revision surgeries compared to vascularized bone flap reconstructions [26]. These results highlight the mechanical and biological advantages of bony reconstruction in reducing hardware complications [26]. More recently, Offodile et al. (2018) compared ALT flaps with plate reconstruction to double free flap reconstructions (combining bone and soft tissue) [18]. Their propensity score-matched study found no significant difference in plate exposure rates between the two groups but noted higher wound infection rates and long-term pain scores in the ALT group [18]. In contrast, Huang et al. (2021) described an increased need for surgical debridement for wound infection in the primary fibula group compared to the primary plate group [20]. Pai et al. (2025) further identified that even in vascularized fibula free flap reconstructions, plate exposure remains a concern, with postoperative radiotherapy and wound infections as independent risk factors [27]. This highlights that while bony flaps offer structural benefits, patient- and treatment-related factors critically influence complication rates [27]. As Onoda et al. (2012) described, plate exposure often results from insufficient soft tissue coverage, mechanical stress, infection, or over-tension at the wound site [28]. In addition, adjuvant radiotherapy further compromises tissue integrity. Ryu et al. (1995) found a clear association between radiation therapy and increased risk of plate exposure and loss due to impaired wound healing [29]. These findings reinforce the need for careful surgical technique, robust flap design, and individualized risk assessment. Strategies to mitigate plate-related complications include selecting flaps with reliable vascularity and bulk, such as ALT, minimizing dead space, avoiding excessive plate bending, and ensuring tension-free closure. In elderly patients with potentially compromised tissue quality and healing capacity, these precautions become even more critical. The relatively high postoperative complication rates observed in the included studies reflect these known risks and underscore the importance of meticulous surgical planning and individualized reconstruction strategy. Additionally, the variability in hospital length of stay may reflect differences in institutional perioperative protocols and patient comorbidity burden.

This review is limited by the small number of eligible studies and sample sizes, the retrospective nature of the data, the variability in outcome reporting, and the multiple sources of heterogeneity in the included studies, which represent considerable methodological constraints. The GRADE assessment of the certainty of evidence showed a very low quality of evidence for all outcomes, suggesting the results should be interpreted very cautiously. The exclusion of non-English language articles might have resulted in language bias, reducing the number of potentially eligible papers. The high reported success rate of ALT flaps might be affected by studies performed in specialized hospitals by experienced and skilled maxillofacial surgery teams and might not reflect results achieved in daily clinical practice. The results of the meta-analyses should be considered cautiously, especially those relative to complications. The only case series study included [19] was judged at high risk of bias, and its exclusion only partially reduced variability across studies. Prediction intervals were estimated to express in an easily interpretable way the amount of heterogeneity in a meta-analysis [37]. Even though their interpretation is straightforward, prediction intervals are strongly based on the assumption of a normal distribution for the effects across studies. Furthermore, when the number of studies is small, they can appear overly wide or overly narrow, and they are recommended when at least five studies are included in a meta-analysis [38], and no clear asymmetry is detected with the funnel plot. Therefore, prediction intervals in the present review should be considered cautiously. Some studies did not report relevant parameters, such as the ASA status, the duration of hospital stay, or details on radiation therapy, making risk stratification or potential subgroup analyses to evaluate the effect of important factors on the results impossible. Another considerable limitation in the literature is that many studies involving soft tissue flaps and plate reconstruction do not specify which flap type was used per patient. The lack of individual data makes it difficult to obtain a precise understanding of the outcomes specifically associated with the ALT flap. Therefore, the true clinical role and efficacy of ALT in this setting may be underestimated or obscured in aggregated data. Nevertheless, it highlights an important clinical niche in which ALT combined with a plate may offer an acceptable compromise between surgical aggressiveness and patient safety. Finally, the patients’ quality of life was addressed only by two of the included studies, using different tools [18,21]. Patient-reported outcome measures are becoming increasingly important in the evaluation of treatment success, especially when the complication rate is not negligible, and hospital stay may last some weeks. It is desirable that these outcomes will be investigated in future studies using standardized questionnaires. Prospective studies with clear documentation of flap type and patient-level data are needed to further define the indications and outcomes of this reconstructive approach in the elderly population.

## 5. Conclusions

In older adults undergoing mandibular reconstruction after ablative surgery, the combination of ALT flap and reconstruction plate offers a less invasive alternative to vascularized bone flaps, especially when these are not applicable because of advanced age and poor prognosis. The ALT flap is the flap of choice when replacing the largest portion of soft tissue removed during the surgical demolition phase. Furthermore, in the absence of a bone component, it is extremely versatile in its three-dimensional positioning, allowing greater freedom of rotation without constraints other than those of the vascular pedicle itself. Depending on the staging, if the site requires adjuvant radiotherapy, thanks to its consistent muscular component, the flap is more resistant to radiation damage. In cases where there is a large bone defect that must be restored during the reconstructive phase, such as reconstruction of the mandibular body, the fibula free flap remains the flap of choice. Only in cases where the latter cannot be used or fails, and titanium osteosynthesis devices must be used to restore the bone defect, such as customized titanium plates, then the ALT flap represents an ideal choice because the flap’s muscular component is extremely effective in covering the titanium component, reducing the risk of exposure and infection. Indeed, the flap’s extended vitality period allows for healing of the overlying tissues, which develop a fibrotic capsule that protects the plaque itself. Depending on any comorbidities associated with the clinical case, the size of the arterial and venous vascular caliber, and the dense vascular network at the muscular level, create the conditions for greater resistance to microcirculatory problems such as diabetes, transient ischemia due to blood pressure changes, or impaired fluid homeostasis due to renal problems. Given the limited and often nonspecific data available, further studies are needed to get robust evidence and better define the role of ALT flaps in the frail population and to guide optimal flap selection.

## Figures and Tables

**Table 1:** Databases searched and search string adapted for each database.

Database	Search String	Number of Records
Pubmed	((“Surgical Flaps” [Mesh]) OR (ALT) OR (anterolateral thigh flap)) AND (“Mandibular Reconstruction” [Mesh]) AND (bone plate)	146
Scopus	(TITLE-ABS-KEY ((surgical flaps) OR (ALT) OR (anterolateral thigh flap)) AND TITLE-ABS-KEY (mandibular reconstruction) AND TITLE-ABS-KEY (bone plate))	385
Web of Science	((((ALL = (surgical flaps)) OR ALL = (ALT)) OR ALL = (anterolateral thigh flap)) AND ALL = (Mandibular Reconstruction)) AND ALL = (bone plate)	184
Embase	(‘surgical flaps’/exp OR ‘surgical flaps’ OR ‘alt’ OR ‘anterolateral thigh flap’/exp OR ‘anterolateral thigh flap’) AND (‘mandibular reconstruction’/exp OR ‘mandibular reconstruction’) AND (‘bone plate’/exp OR ‘bone plate’)	121

**Table 2:** Demographic and clinical characteristics of patients undergoing ALT flap and plate mandibular reconstruction.

Characteristic	Bowe et al. 2021 [19]	Chang et al. 2023 [21]	Offodile et al. 2018 [18] **	Huang et al. 2021 [20]
Number of Patients	30	58	127	114
Age (years), mean value ± SD (range)	67 (31–87)	56.8 *	55.9 ± 12.3	56.3 ± 10
Gender (Male/Female)	19/11	56/2	120/7	105/9
Primary Diagnosis	Malignancy: 28 (91.6%) Osteoradionecrosis: 2 (8.3%)	Malignancy: 57 (98.3%) Osteoradionecrosis: 1 (1.7%)	Malignancy: 127 (100%)	Malignancy: 114 (100%)
Malignancy stage	IV: 26 Recurrent: 2	Early (I, II): 6 Advanced (III, IV): 51	T2/T3: 13 T4a: 93 T4b: 21	I: 5 II: 8 III: 3 IV: 98
Physical Status (ASA classification)	ASA 1: 10 ASA 2: 18 ASA 3: 2	NR	NR	NR
Defect Location	Posterior and lateral anterior mandible	Posterior and lateral anterior mandible	Posterior and lateral anterior mandible	Posterior mandible
length of Hospital Stay (days), mean ± SD	10 *	19.5 *	23.9 ± 11.6	NR

* median; ** 31 of 127 patients were selected for propensity score-matched study; ASA: American Society of Anaesthesiologists; SD: standard deviation; NR: not reported.

**Table 3:** Postoperative Complications Related to Flap, Plate, and Surgical Wound.

Complication Type	Bowe et al. 2021 [19]	Chang et al. 2023 [21]	Offodile et al. 2018 [18] *	Huang et al. 2021 [20]
Flap survival/Complications	Flap loss: 1 (3.3%)	Partial necrosis: 4 (6.6%) Flap loss: 3 (5.2%)	No flap loss	No flap loss
Plate Complications	Plate-related (total): 3 (10%) Plate infection: 2 (6.7%) Plate extrusion: 1 (3.3%)	Plate-related (total): 22 (37.9%) ** Plate exposure: 14 (24.6%) Plate fracture/dislodgement: 14 (24.6%)	Plate exposure: 12 (38.7%) Plate fracture: 1 (3.2%)	Plate exposure: 31 (27%)
Surgical Wound Complications	NR	Infection 20.7% Fistula 31%	Wound infection/fistula: 12 (38.7%)	NR

* Results are reported for the cohort of 31 patients selected for the propensity score-matched study; ** some cases with plate exposure also had plate fracture/dislodgement and were counted twice; NR: not reported.

## Data Availability

No new data were created or analyzed in this study. Data sharing is not applicable to this article.

## Supplementary Materials

The following supporting information can be downloaded at https://www.mdpi.com/article/10.3390/jcm15093457/s1. Table S1: Assessing the certainty of evidence across studies for an outcome. Checklist [39].
