# Supplementary material for: Anterolateral Thigh Flap and Bone Plate for Mandibular Reconstruction in Patients over 55 Undergoing Ablative Oral Surgery: A Systematic Review and Meta-Analysis
Source: J Clin Med. 2026 May 1;15(9):3457. doi: 10.3390/jcm15093457 (PMC13163595; doi:10.3390/jcm15093457)
Supplement: Supplementary file 1 [file jcm-15-03457-s001.zip › jcm-4200230-supplementary-table.pdf]

GRADE summary Table S1: Assessing the certainty[1] of evidence across studies for an outcome

Anterolateral Thigh Flap and bone Plate for Mandibular Re-construction in Patients Over 55 Undergoing Ablative Oral Surgery

Certainty assessment of evidence for each outcome

No of studies	Design	Risk of bias	Inconsistency	Indirectness[2]	Imprecision	Other[3]	Relative effect (95% CI)	Certainty (overall score)[4]
	**Outcome:** flap success rate, %							
4 (total 233 patients)	retrospective (3 cohort, 1 case series) (-0.5)	Serious risk of bias (-0.5)	fair inconsistency (-0.5)	No serious indirectness (N.A. for case series	No serious imprecision	Different gender ratio, follow-up duration; malignancy stages and defect location (-0.5)	Summarized proportion 0.99 (0.94, 1.00)	Very low (1) ⊕◯◯◯
	**Outcome:** plate-related complication rate, %							
4 (total 233 patients)	retrospective (3 cohort, 1 case series) (-0.5)	Serious risk of bias (-0.5)	Important inconsistency (-0.5)	No serious indirectness (N.A. for case series	No serious imprecision	Different gender ratio, follow-up duration; malignancy stages and defect location (-0.5)	Summarized proportion 0.28 (0.15, 0.40)	Very low (1) ⊕◯◯◯
	**Outcome:** overall complication rate, %							
4 (total 233 patients)	retrospective (3 cohort, 1 case series) (-0.5)	Serious risk of bias (-0.5)	Important inconsistency (-0.5)	No serious indirectness (N.A. for case series	No serious imprecision	Different gender ratio, follow-up duration; malignancy stages and defect location (-0.5)	Summarized proportion 0.52 (0.26, 0.78)	Very low (2) ⊕◯◯◯

[1] This can also be referred to as 'quality of the evidence' or 'confidence in the estimate'. The "certainty of the evidence" is an assessment of how good an indication the research provides of the likely effect; i.e. the likelihood that the effect will be substantially different from what the research found. By "substantially different" we mean a large enough difference that it might affect a decision.

[2] Indirectness includes consideration of

- Indirect (between study) comparisons
- Indirect (surrogate) outcomes
- Applicability (study populations, interventions or comparisons that are different than those of interest)

[3] Other considerations for downgrading include publication bias. Other considerations for upgrading include a strong association with no plausible confounders, a dose response relationship, and if all plausible confounders or biases would decrease the size of the effect (if there is evidence of an effect), or increase it if there is evidence of no harmful effect (safety)

[4] 4 ⊕⊕⊕⊕ **High** = This research provides a very good indication of the likely effect. The likelihood that the effect will be substantially different** is low.
3 ⊕⊕⊕◯ **Moderate** = This research provides a good indication of the likely effect. The likelihood that the effect will be substantially different** is moderate.
2 ⊕⊕◯◯ **Low** = This research provides some indication of the likely effect. However, the likelihood that it will be substantially different** is high.
1 ⊕◯◯◯ **Very low** = This research does not provide a reliable indication of the likely effect. The likelihood that the effect will be substantially different** is very high.

** Substantially different = a large enough difference that it might affect a decision
